# The Genetic Background of Hearing Loss in Patients with EVA and Cochlear Malformation

**DOI:** 10.3390/genes14020335

**Published:** 2023-01-28

**Authors:** Natalia Bałdyga, Dominika Oziębło, Nina Gan, Mariusz Furmanek, Marcin L. Leja, Henryk Skarżyński, Monika Ołdak

**Affiliations:** 1 Department of Genetics, Institute of Physiology and Pathology of Hearing, 02-042 Warsaw, Poland; 2 Doctoral School of Translational Medicine, Medical Centre of Postgraduate Education, 01-813 Warsaw, Poland; 3 Bioimaging Research Center, Institute of Physiology and Pathology of Hearing, 02-042 Warsaw, Poland; 4 Oto-Rhino-Laryngology Surgery Clinic, Institute of Physiology and Pathology of Hearing, 02-042 Warsaw, Poland

**Keywords:** hearing loss, inner ear malformation, enlarged vestibular aqueduct (EVA), incomplete partition type 2 (IP2), high-throughput sequencing, pathogenic variants

## Abstract

The most frequently observed congenital inner ear malformation is enlarged vestibular aqueduct (EVA). It is often accompanied with incomplete partition type 2 (IP2) of the cochlea and a dilated vestibule, which together constitute Mondini malformation. Pathogenic *SLC26A4* variants are considered the major cause of inner ear malformation but the genetics still needs clarification. The aim of this study was to identify the cause of EVA in patients with hearing loss (HL). Genomic DNA was isolated from HL patients with radiologically confirmed bilateral EVA (*n* = 23) and analyzed by next generation sequencing using a custom HL gene panel encompassing 237 HL-related genes or a clinical exome. The presence and segregation of selected variants and the CEVA haplotype (in the 5′ region of *SLC26A4*) was verified by Sanger sequencing. Minigene assay was used to evaluate the impact of novel synonymous variant on splicing. Genetic testing identified the cause of EVA in 17/23 individuals (74%). Two pathogenic variants in the *SLC26A4* gene were identified as the cause of EVA in 8 of them (35%), and a CEVA haplotype was regarded as the cause of EVA in 6 of 7 patients (86%) who carried only one *SLC26A4* genetic variant. In two individuals with a phenotype matching branchio-oto-renal (BOR) spectrum disorder, cochlear hypoplasia resulted from *EYA1* pathogenic variants. In one patient, a novel variant in *CHD7* was detected. Our study shows that *SLC26A4*, together with the CEVA haplotype, accounts for more than half of EVA cases. Syndromic forms of HL should also be considered in patients with EVA. We conclude that to better understand inner ear development and the pathogenesis of its malformations, there is a need to look for pathogenic variants in noncoding regions of known HL genes or to link them with novel candidate HL genes.

## 1. Introduction

In subjects with congenital hearing loss (HL), radiological data show that inner ear malformations (IEMs) occur in about 20% of them [1]. The majority of these patients have bilateral severe to profound HL. Although it might seem that these subjects could benefit from a cochlear implant (CI), surgical placement of a CI can sometimes be challenging or impossible due to severe IEMs. However, in the future other treatments may become available, so it is important to better understand the role of genes in inner ear development. 

IEMs may be identified in patients with isolated or syndromic HL [2]. IEMs are one of the characteristic features of genetically determined syndromes such as Pendred syndrome [3], branchio-oto-renal syndrome (BOR) [4], Waardenburg syndrome [5], or CHARGE [6]. The most frequent IEM detected during radiological imaging of HL patients is enlarged vestibular aqueduct (EVA) [7,8,9]. The combination of EVA, enlarged vestibule, and incomplete cochlear partition (limited to its upper part and known as incomplete partition type 2, IP2) has been named Mondini malformation [10]. In IP2, the middle and apical turns of the cochlea coalesce to form a cystic apex [11,12].

Typically, HL in EVA/IP2 patients is prelingual and severe to profound, but it may also have a later onset and be progressive or fluctuating as well as variable in severity [13,14]. In some patients, there may be a low-frequency air-bone gap resulting from a third window effect, where the connection of the vestibular aqueduct to the vestibule is larger than normal and acts as a third window which leaks acoustic energy from the aqueduct to the dura. The third window effect creates a conductive HL [12,15].

According to the literature, the most common cause of HL involving EVA/IP2 are recessive variants in the *SLC26A4* gene [16,17]. Pathogenic variants of *SLC26A4* lead to the development of DFNB4 and Pendred syndrome, in which HL is accompanied by abnormal organification of iodine, which leads to development of goiter with or without hypothyroidism. *SLC26A4* encodes the transmembrane anion exchanger pendrin, which is predominantly expressed in the inner ear, thyroid, kidney, and airway epithelia [18,19,20,21,22]. In the inner ear pendrin is essential for maintaining ion homeostasis and the endolymphatic potential [23,24]. Pendrin is also crucial for the development of the auditory and vestibular system, with loss of pendrin leading to degeneration of hair cells [25].

In the Caucasian population, approximately 25% patients with EVA/IP2 and HL have biallelic pathogenic variants of *SLC26A4* (M2) and another 25% of patients carry a monoallelic *SLC26A4* variant (M1) [26,27,28]. In the remaining 50% of subjects, no pathogenic variants in the coding and splice site regions of *SLC26A4* are found (M0) [29]. Some studies suggest that in M1 patients an unidentified variant may be present in the *trans* configuration with a defective *SLC26A4* allele [29]. Supporting that interpretation, Chattaraj et al. reported a haplotype containing 12 single nucleotide polymorphisms (SNPs) spanning a 613 kb upstream region of the *SLC26A4* gene. The Caucasian EVA (CEVA) haplotype was identified in 13/16 studied M1 subjects and was enriched in the M0 group [28].

In this study, we performed audiological evaluation and genetic testing by next generation sequencing (NGS) using a custom HL gene panel or a clinical exome of 23 patients with bilateral IEM confirmed radiologically by the same radiologist. A newly identified *SLC26A4* synonymous variant was functionally tested using a minigene assay. The presence of a CEVA haplotype was also analyzed. This study provides further insight into the genetic background of EVA/IP2 and its genotype–phenotype correlations.

## 2. Materials and Methods

### 2.1. Study Subjects

A group of 23 Polish patients with radiologically confirmed bilateral IEM and HL was recruited to the study. High resolution computed tomography (CT) of the temporal bone was performed using a slice thickness of 0.4 mm (SOMATOM Definition AS, Siemens, Munich, Germany). Image stacks were analyzed by a single experienced radiologist on a professional workstation (syngo.via, Siemens, Munich, Germany) using a multiplanar reconstruction technique. IEMs were recognized based on previously published criteria [10,11,30], from which all 23 patients were deemed to have EVA. In 10 cases, EVA was the only abnormality. In 11 patients EVA was accompanied with IP2 but with no semicircular canal malformation. In 2 patients, in addition to EVA, cochlear hypoplasia type 4 (CH4) was recognised together with severe hypoplasia of the posterior semicircular canal (reduced to ampulla). All patients were prescreened for pathogenic variants at the DFNB1 locus and had no risk factors for HL development. Patients or their legal guardians signed informed consent forms. The study was approved by the ethics committee at the Institute of Physiology and Pathology of Hearing (KB.IFPS.05/2022) and performed according to the Declaration of Helsinki.

### 2.2. Genetic Testing

Probands’ DNA was isolated from whole blood samples using standard salting out procedure. From available family members, buccal swabs were obtained, and genomic DNA was isolated with the automatic method on a Maxwell RSC instrument (Promega, Walldorf, Germany) according to the manufacturer’s protocol. For 21 probands, custom multigene panel testing which included 237 HL-related genes was performed (SeqCap EZ Choice Probes, Roche, Basel, Switzerland), and enriched libraries were further run with the MiSeq Reagent Kit V3 (150 cycles, 2 × 75 bp). For 2 patients, clinical exome sequencing was performed (TruSightOne Sequencing Panel, Illumina, San Diego, CA, USA) and libraries were sequenced with the MiSeq Reagent Kit V3 (300 cycles, 2 × 150 bp) and the MiSeq instrument (Illumina, San Diego, CA, USA).

Bioinformatics and expert analyses were performed as previously described [31]. The pathogenicity of identified variants was assigned based on ACMG/AMP Interpreting Sequence Guidelines [32] with specifications for HL [33]. Variants were annotated with the Ensembl VEP [34], as well as dbSNP [35] or dbNSFP [36], and classified using allele frequency (Genome Aggregation Database, gnomAD, https://gnomad.broadinstitute.org; UK10K, https://www.uk10k.org; Exome Variant Server, EVS; https://evs.gs.washington.edu/EVS; all accessed on 1 December 2022), in silico predictions (PolyPhen-2, SIFT, Mutation Taster, LRT, and CADD), annotations from public variant databases (ClinVar, HGMD), matches from an in-house variants database, and information from related medical literature.

The presence of probably causative variants (pathogenic, probably pathogenic, or VUS) was confirmed, and detection of CEVA haplotype and segregation analyses were performed using standard Sanger sequencing. Primer sequences are available upon request.

### 2.3. Minigene Splicing Assay

Sequences containing exons 1–3 of the *SLC26A4* gene, and flanking intronic regions upstream (~100 bp) and downstream (~1500 bp), were amplified from genomic DNA with Phusion High-Fidelity DNA Polymerase (Thermo Fisher Scientific, Waltham, MA, USA) using a two-step protocol. This region was then cloned to expression vector pDEST pCI-NEO RHO (kindly provided by Professor F. P. M. Cremers, Radboud University, the Netherlands) with Gateway cloning technology using pDONR221 as the transient donor vector [37]. The variant of interest (*SLC26A4* c.162C>T) was introduced into the expression vector containing a wild type sequence using directional mutagenesis (QuikChange II Site-Directed Mutagenesis Kit, Agilent Technologies, Santa Clara, CA, USA). After each step, the sequence of the insert was verified using Sanger sequencing.

HEK293T cells (ATCC Manassas, VA, USA) were transfected with both constructs and pDEST pCI-NEO RHO empty vector (5000 ng each) in triplicates using Lipofectamine 3000 Transfection Reagent (Thermo Fisher Scientific, Waltham, MA, USA) according to the standard protocol. Cells were harvested after 48 h incubation and total RNA was isolated using a RNeasy Mini Kit (Qiagen, Hilden, Germany). RNA (250 ng) underwent reverse transcription using an RevertAid First Strand cDNA Synthesis Kit (Thermo Fisher Scientific, Waltham, MA, USA) and *RHO* gene-specific primer 5′CACCTGGCTCG 3′. Generated cDNA was used as matrix in PCR and analyzed with gel electrophoresis and Sanger sequencing.

### 2.4. Clinical Evaluation

Patients’ medical records were analyzed. Thyroid status, age at HL onset, as well as data on use of hearing aids (HAs) or CIs, were collected. All available pure-tone audiometry (PTA) results of the probands were gathered and analyzed. HL was referred to as asymmetric when a difference of ≥15 dB between right and left ears occurred. To calculate HL severity for single ears with EVA, the last air-conduction thresholds for frequencies from 0.5 to 4 kHz preceding CI surgery were used (except for patients 7599 and 8078 who were implanted before 5 y.o.) [38]. When there was no response to an air-conducted stimulus, a value of 125 dB was used. Analyses of differences between patient groups with different genotypes were performed using a nonparametric Mann–Whitney test. Statistical analyses were performed using GraphPad Prism 5 Software (La Jolla, CA, USA).

## 3. Results

### 3.1. Importance of SCL26A4 Variants in Development of IEMs

Genetic testing revealed causative variants in 74% (17/23) of the probands. In almost half of them (47%; 8/17), two pathogenic variants located on both alleles of the *SLC26A4* gene were identified (M2 group) (Table 1 and Table 2). In one patient from this group (8078), a homozygous CEVA haplotype was also present (included in the M2 group). In 7 consecutive patients, only one pathogenic variant in the *SLC26A4* gene was detected (M1 group) and in 6 of them it was accompanied with a complete CEVA haplotype on the second allele (M1 + CEVA group) (Table 1 and Table 2). In this group, one patient (4963) was also homozygous for the CEVA haplotype (included in the M1 + CEVA group). Among the pathogenic variants detected in the *SLC26A4* gene, the majority were known variants (80%; 12/15) which had been previously described in the literature as causative of Pendred syndrome or isolated HL (DFNB4). The most frequently identified variant was *SLC26A4* c.85G>C (p.Glu29Gln). In the remaining 8 probands, no pathogenic variants in the *SLC26A4* gene were identified (M0 group) (Table 1 and Table 2). Analysis of the results showed another syndromic form of HL in two of the tested patients. Respectively, they carried novel c.1475+1G>T (p.?) or c.1329_1330del (p.Glu443AspfsTer8) pathogenic variants in the *EYA1* gene, which is involved in the development of BOR spectrum disorder. In one patient, a variant of unknown significance (c.4477C>T; p.Arg1493Cys) in the *CHD7* gene was identified (Table 2). 

### 3.2. Pathogenic Character of the SLC26A4 c.162C>T Synonymous Variant

In patient 7966, a novel synonymous *SLC26A4* c.162C>T variant was detected. Based on ACMG/AMP criteria, it was first classified as a variant of unknown significance (VUS). However, the SpliceAI algorithm indicated its possible impact on splicing. Detailed bioinformatics analysis predicted (i) loss of the natural donor site (SSF and NNSPLICE) and loss of the second exon, or (ii) weakness of the natural site, creation of alternative donor site at c.160, and, in consequence, shortening of the transcript by four nucleotides (MaxEnt and GeneSplicer).

**Figure 1:**
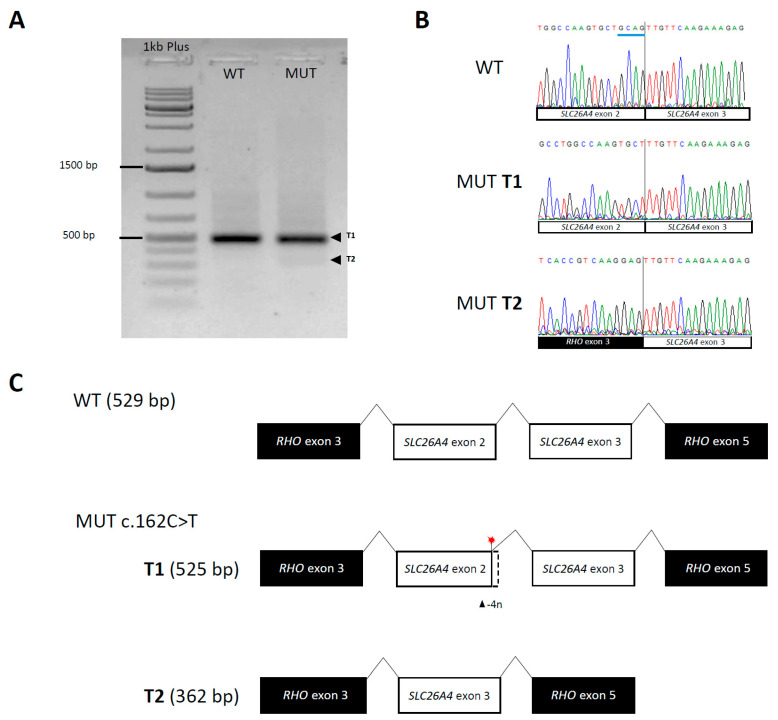
Results of minigene assay for the *SLC26A4* c.162C>T variant. Introduction of the c.162C>T variant resulted in creation of two mutated transcripts—T1 with novel donor site at c.160 and shortened by four nucleotides, and T2 lacking exon 2. (**A**) The *SLC26A4* transcripts visualized with gel electrophoresis. (**B**) Results of the Sanger sequencing of wild type and mutated transcript. (**C**) Schematic representation of wild type and mutated transcripts. WT, wild type; MUT, mutant; T1, transcript 1; T2, transcript 2; bp, base pairs.

Using minigene assay, these hypotheses were tested and both scenarios were confirmed. The majority of mutated transcripts were shortened by four nucleotides (T1, Figure 1). There was also a small fraction of transcripts lacking exon 2 (T2, Figure 1). As a result, mutated transcripts are predicted to produce shortened proteins, i.e., p.Cys54PhefsTer11 and p.Met1?.

**Table 2:** List and characteristics of the probably causative variants which were identified.

Proband ID	Gene	Variant cDNA Level	Variant Protein Level	General Population Frequencies			Pathogenicity Predictions						ACMG Classification	HGMD Accession No.
				gnomAD	UK10K	EVS	SIFT	Poly-Phen-2	Mutation Taster	LRT	CADD	SpliceAI		
60	*SLC26A4*	c.412G>T	p.Val138Phe	0.00018	0.00026	0.00008	D	D	D	D	22.9	–	LP	CM981497
		c.1001+1G>A	p.?	0.00024	0.00026	0.00046	–	–	D	–	26.1	A	P	CS982315
88	*SLC26A4*	c.1262A>G	p.Gln421Arg	0.00002	0	0.00008	D	D	D	D	26.9	A	LP	CM040114
		het CEVA haplotype												
4314	no causative variants													
4963	*SLC26A4*	c.2219G>T	p.Gly740Val	0.00023	0.00013	0.00015	D	T	D	D	23.2	–	LP	CM061995
		hom CEVA haplotype												
7599	*SLC26A4*	c.707T>C	p.Leu236Pro	0.00034	0.00053	0.00069	D	D	D	T	28.8	–	LP	CM981500
		c.1001+1G>A	p.?	0.00024	0.00026	0.00046	–	–	D	–	26.1	A	P	CS982315
7966	***SLC26A4***	**c.162C>T**	**p.Cys54PhefsTer11; p.Met1?**	**0**	**0**	**0**	**–**	**–**	**–**	**–**	**21.3**	**A**	**LP**	**–**
		het CEVA haplotype												
9172	*SLC26A4*	c.85G>C	p.Glu29Gln	0.00011	0	0	T	D	D	D	23.1	–	P	CM011487
		het CEVA haplotype												
10465	no causative variants													
11950	*SLC26A4*	c.1003T>C	p.Phe335Leu	0.00079	0.00130	0.00120	T	D	D	D	24.3	–	LP	CM011490
11994	***SLC26A4***	c.1246A>C	p.Thr416Pro	0.00021	0.00026	0.00015	D	D	D	D	26.8	–	LP	CM981505
		**c.1708G>A**	**p.Val570Ile**	**0.00006**	**0**	**0**	**T**	**D**	**D**	**D**	**25.6**	**–**	**LP**	**–**
900	***CHD7***	**c.4477C>T**	**p.Arg1493Cys**	**0.00001**	**0**	**0**	**D**	**D**	**D**	**D**	**34**	**–**	**VUS**	**–**
4655	***SLC26A4***	**c.284G>A**	**p.Gly95Glu**	**0**	**0**	**0**	**D**	**D**	**D**	**D**	**28.3**	**–**	**LP**	**–**
		c.1262A>G	p.Gln421Arg	0.00002	0	0.00008	D	D	D	D	26.9	A	LP	CM040114
5013	***EYA1***	**c.1475+1G>T**	**p.?**	**0**	**0**	**0**	**–**	**–**	**D**	**–**	**26.5**	**A**	**P**	**–**
5149	***SLC26A4***	c.1229C>T	p.Thr410Met	0.00004	0.00013	0.00023	D	D	D	D	33	–	LP	CM981504
		**c.1670G>T**	**p.Gly557Val**	**0**	**0**	**0**	**D**	**D**	**D**	**D**	**26.7**	**–**	**LP**	**–**
6713	no causative variants													
8078	*SLC26A4*	c.85G>C	p.Glu29Gln	0.00011	0	0	T	D	D	D	23.1	–	P	CM011487
		c.845G>A	p.Cys282Tyr	0.00001	0	0.00008	D	D	D	D	27.7	–	LP	CM165413
		hom CEVA haplotype												
13358	*SLC26A4*	c.85G>C	p.Glu29Gln	0.00011	0	0	T	D	D	D	23.1	–	P	CM011487
		het CEVA haplotype												
13362	*SLC26A4*	c.1262A>G	p.Gln421Arg	0.00002	0	0.00008	D	D	D	D	26.9	A	LP	CM040114
		het CEVA haplotype												
13392	no causative variants													
13393	no causative variants													
13887	***EYA1***	**c.1329_1330del**	**p.Glu443AspfsTer8**	**0**	**0**	**0**	**–**	**–**	**–**	**–**	**–**	**A**	**P**	**–**
4319	*SLC26A4*	c.85G>C	p.Glu29Gln	0.00011	0	0	T	D	D	D	23.1	–	P	CM011487
		c.2027T>A	p.Leu676Gln	0	0	0	T	D	D	D	24.6	–	LP	CM030963
11262	*SLC26A4*	c.412G>T	p.Val138Phe	0.00018	0.00026	0.00008	D	D	D	D	22.9	–	LP	CM981497
		c.707T>C	p.Leu236Pro	0.00034	0.00053	0.00069	D	D	D	T	28.8	–	LP	CM981500

Novel variants are bolded. Het, heterozygous; hom, homozygous; P, pathogenic; LP, likely pathogenic; VUS, variant of unknown significance; D, deleterious; T, tolerated; A, affects; ‘–’, not applied. Reference sequences: *CHD7* NM_017780.4 and NP_060250.2, *EYA1* NM_000503.6 and NP_000494.2, *SLC26A4* NM_000441.2 and NP_000432.1.

### 3.3. Genotype-Dependent Differences in Clinical Pictures

Reanalysis of the patients’ clinical data in the context of their genetic background, IEMs, and HL demonstrated some genotype–phenotype correlations. CT imaging revealed more severely affected inner ears in patients from the M2 group. All of them had bilateral EVA accompanied with dysplasia of mid-apical parts of the cochlea (IP2). In this group, the median age of HL onset was 0.75 y.o., significantly lower than the 6 y.o. in the M1 + CEVA group (*p* < 0.01). The median age of HL onset in the M1 and M0 groups was 4 y.o. (Table 1). In the majority of probands, HL was asymmetric and fluctuating. All patients were fitted with HAs or implanted with CIs. In almost all patients (6/8) from the M2 group, goiter and/or hypothyroidism were observed, indicating a diagnosis of Pendred syndrome. Similar thyroid signs were observed only sporadically in the M1 + CEVA and M0 groups (Table 1). 

**Figure 2:**
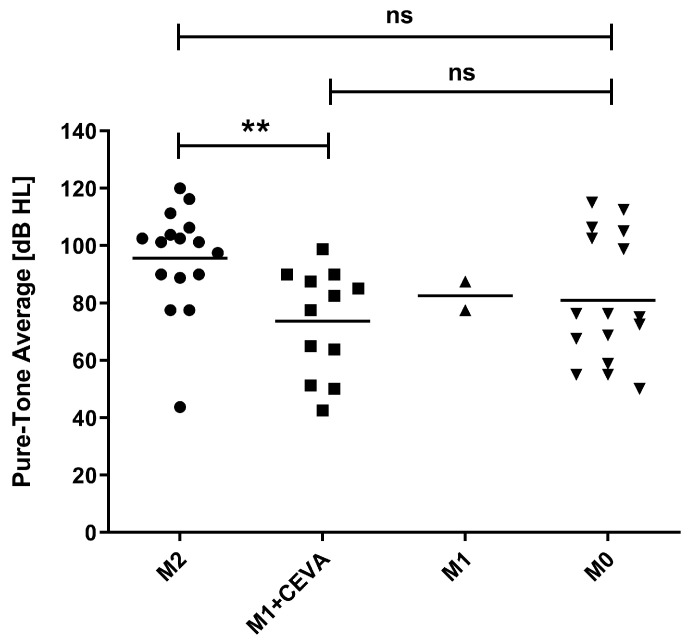
Average air-conduction thresholds for ears with EVA malformation. Each point represents one ear displayed according to *SLC26A4* genotype. M2 indicates patients with two pathogenic variants of *SLC26A4*; M1 + CEVA indicates patients with one pathogenic variant of *SLC26A4 in trans* configuration with the CEVA haplotype; M1 indicates patients with only one *SLC26A4* pathogenic variant; M0 indicates patients with no pathogenic variants in the *SCL26A4* gene and the CEVA haplotype; ns, non-significant; **, *p* ≤ 0.01.

The median air-conduction threshold in the M2 group was 101.3 dB, statistically significantly higher than 80.0 dB in patients from the M1 + CEVA group (*p* < 0.01). There was no statistically significant difference between the median thresholds of the M2 and M0 patients (101.3 dB vs. 75.6 dB; *p* = 0.068). Patients from the M1 + CEVA and M0 groups had comparable median air-conduction thresholds (82.5 dB vs. 73.8 dB, *p* = 0.84) (Figure 2).

## 4. Discussion

In this study we searched HL patients for the molecular basis of isolated EVA or EVA accompanied with cochlear malformation, and verified the hypothesis of an association between the number of *SLC26A4* pathogenic variants and the severity of HL. We found causative variants of the *SLC26A4* gene in over 60% of patients, and detailed genotype–phenotype analyses showed a higher incidence of thyroid enlargement/malfunction and earlier and more severe HL in patients with two pathogenic variants located in *SLC26A4* (M2) compared to those carrying only one pathogenic variant in the *SLC26A4* gene *in trans* configuration with the CEVA haplotype (M1 + CEVA). Identification of several novel variants, not previously described in the context of any disease, expands our knowledge of genetic determinants of IEMs and confirms the need to use high-throughput sequencing methods when genetically diagnosing patients with IEMs. 

Despite technological breakthroughs and the introduction of high-throughput sequencing methods for diagnosing patients with HL and IEMs, more than half the cases still remain unsolved [39,40]. Our results align with studies conducted in North American Caucasian and European EVA patients [28,41]. In almost 25% of patients with EVA, two pathogenic variants are usually identified; in 25%, only one pathogenic variant of this gene is reported; in the remaining 50% there are no causative variants in *SLC26A4* [26,29,42]. This differs markedly from Asian populations, where pathogenic variants in *SLC26A4* are identified much more frequently (up to 90% of patients [43,44,45]), findings which strongly suggest that there must be specific variants in the Caucasian population that are being missed with standard diagnostic approaches. 

The discovery by Griffith and colleagues in 2017 of a set of twelve SNPs located in the 5′ region of the *SLC26A4* gene (CEVA haplotype) has increased the detection rate of causative variants when analyzed as probably pathogenic *in trans* configuration with pathogenic variants in the coding and splicing regions of the *SLC26A4* gene [28]. In the literature, the CEVA haplotype is present in 70% (7/10) of patients from a North American Caucasian population, as well as in all (6/6) Danish patients and in 63% (10/16) of Dutch patients with monoallelic variants in *SLC26A4* [28,41]. Our results are similar, with the CEVA haplotype identified in 86% (6/7) of monoallelic *SLC26A4* patients. The higher incidence of this haplotype in EVA patients compared to control individuals strongly supports its possible pathogenic role; however, there is still no functional link between the CEVA haplotype and the development of EVA and HL. There is a need to expand the search for hidden true pathogenic variants of the *SLC26A4* gene to include noncoding regions of the gene, particularly in deep intronic and regulatory regions. It is possible that the missing causative variants are in linkage disequilibrium and co-segregate with the CEVA haplotype, or perhaps some of its 12 SNPs are located in cochlea-specific *SLC26A4* regulatory regions. In some patients a shortened version of the CEVA haplotype was identified, and this finding reduces the region of interest by the first three SNPs. Further research on the genetic background of EVA should consider functional characterization of *SLC26A4* genomic architecture in a cell-specific manner [41].

Another possible avenue for increasing the detection rate of genetic causes of EVA, one which has been postulated in previously published papers, is digenic inheritance of one pathogenic variant in *SLC26A4* and one pathogenic variant in *FOXI1*, *KCNJ10*, or *EPHA2* [46,47,48]. Both *FOXI1* and *EPHA2* are genes encoding proteins interacting with the *SLC26A4* gene or its protein product, pendrin. FOXI1 is a transcription factor binding to the *SLC26A4* promotor region and regulating its transcriptional activity. Pathogenic variants of this gene have been identified in monoallelic *SLC26A4* patients; however, possible digenic inheritance has not been confirmed [49,50,51,52]. Despite extensive functional studies and proof that EPHA2 is a molecular partner of SLC26A4, there is insufficient evidence for digenic inheritance of *EPHA2* and *SLC26A4* gene variants. Pathogenic variants of *EPHA2* have so far been identified in only two Japanese monoallelic *SLC26A4* patients [48]. The *KCNJ10* gene encodes inwardly rectifying K^+^ channel subunit abundantly expressed in the stria vascularis. In the Slc26a4 –/– mouse, a reduction of Kcnj10 protein level is observed, which suggests an indirect relationship between these proteins [24,53]. Up to now, pathogenic variants in the *KCNJ10* gene have been identified only sporadically in monoallelic *SLC26A4* patients [49,50,51,52] and there are no data confirming digenic inheritance of *KCNJ10* and *SLC26A4* pathogenic variants.

In our study we did not identify pathogenic variants in *FOXI1* and *KCNJ10* genes. Our multigene panel testing did not include the *EPHA2* gene. Genetic testing did reveal that novel pathogenic variants of the *EYA1* gene were involved in the development of IEMs. Reanalysis of patients’ clinical data confirmed a diagnosis of BOR spectrum disorder. Cochlear hypoplasia type 4 is one of the most frequently identified CT findings in BOR patients [4,54,55]. In one patient, a c.4477C>T (p.Arg1493Cys) variant, classified as VUS, was detected in the *CHD7* gene. This variant was not present in population databases, but different computational algorithms predicted that it is pathogenic. Unfortunately, there were no available family members to perform segregation analysis, so there is a need to perform clinical reanalysis of patient phenotype and obtain additional evidence to unequivocally confirm pathogenicity of the identified variant. IEMs have been previously described in patients with CHARGE syndrome caused by mutations in the *CHD7* gene. Typical radiological imaging shows cochlear hypoplasia and bony cochlear nerve canal hypoplasia in association with absence of the semicircular canals [56]. Some of the identified variants in the *CHD7* gene are associated with a milder clinical picture of CHARGE syndrome, and it cannot be ruled out that in the case of the examined patient the identified variant causes only EVA. 

So far, many studies have addressed the issue of the relationship between the genetic background of inner ear malformations and the clinical picture of patients, in particular the number or type of genetic variants and the severity of HL [38,41,44,57]. It has been repeatedly shown that patients with biallelic *SLC26A4* variants (M2) have an earlier age of HL onset, their HL is more severe, and they are more often diagnosed with Pendred syndrome than patients with a monoallelic *SLC26A4* variant and CEVA (M1 or M1 + CEVA) or without pathogenic variants in the *SLC26A4* gene (M0) [26,58,59]. In recent years, contradictory results have been presented in two studies, one conducted in North American Caucasian patients and another in Dutch patients. Chao et al. observed thyroid dysfunction and more severe HL in patients from an M2 group (median 86.3 dB HL) compared to M1 + CEVA patients (median 47.5 dB HL) [38]. This finding was not replicated in the study by Smits et al., where no significant differences were observed between the median HL in patients from the M2 (84 dB HL) or M1 + CEVA (85 dB HL) groups [41]. A possible explanation for this discrepancy is a difference in HL progression and average age between the cohorts. Our results agree with those from the study by Chao et al., in that we observed significantly more severe HL in M2 patients (median 101.3 dB HL) than in patients from the M1 + CEVA group (median 80 dB HL). However, hearing thresholds, especially in the M1 + CEVA group, more resembled those reported in the Dutch study. It is possible that severe HL in the Polish patients may be the result of more severe cochlear malformations. In this study all patients had bilateral inner ear malformation, and in 48% of them EVA and IP2 were identified. Previous reports have focused on patients who, mainly or exclusively, had bilateral or unilateral EVA. Another explanation is a possible relationship between the type of identified variant and HL severity, but up to now this has only been reported for Korean patients, where the authors showed that HL progressed more frequently in patients with homozygous missense variant (p.His723Arg/p.His723Arg) than in patients with compound heterozygous missense and splice-site variants (p.His723Arg/c.919-2A>G) [57]. 

The major limitation of our study is the size of the selected group of patients. A larger, homogenous group of patients with IEMs and the use of more comprehensive genetic testing methods may provide stronger evidence for the observed genotype–phenotype correlation. Large multicenter studies are needed to expand knowledge and fully characterize the link between the molecular background of IEMs and the clinical pictures of the patients.

## 5. Conclusions

In this study we performed multigene panel testing in patients with bilateral isolated EVA or EVA accompanied with cochlear malformation. We detected high rates of probably causative variants and identified novel variants that have not been previously linked to the disease. These findings expand our current state of knowledge on the genetic background of IEMs and provide additional evidence of genotype–phenotype correlation in patients with *SLC26A4* pathogenic variants. The work confirms that patients with biallelic *SLC26A4* mutations have more severe HL at an earlier age of onset.

## Figures and Tables

**Table 1:** Clinical data of tested patients and their assignment to groups based on the *SLC26A4* genotype.

Proband ID	Group	IEM	Thyroid Problems	Age at HL Onset [y.o.]	Age at Examination [y.o.]	HL Symmetry	HA/CI
60	M2	IP2+EVA	+	congenital	28	+	HAs
88	M1+CEVA	IP2+EVA	+	3	32	+	HAs
4314	M0	EVA	–	5	48	–	HAs
4963	M1+CEVA	EVA	–	6	18	–	CI RE 18 y.o.
7599	M2	IP2+EVA	+	congenital	9	–	CI RE 3 y.o.; CI LE 4 y.o.
7966	M1+CEVA	EVA	–	3	17	–	HAs
9172	M1+CEVA	IP2+EVA	–	8	26	–	HAs
10465	M0	EVA	+	2.5	21	–	CI RE 21 y.o.
11950	M1	IP2+EVA **	–	4	22	+	HAs
11994	M2	IP2+EVA **	–	3	21	–	CI LE 19 y.o.
900	M0	EVA	–	3	21	–	CI LE 22 y.o.
4655	M2	IP2+EVA	–	1.5	10	+	CI RE 10 y.o.
5013	M0	EVA+other *	–	1	15	–	CI RE 15 y.o.
5149	M2	IP2+EVA	+	5	41	+	CI LE 43 y.o.
6713	M0	EVA **	–	10	35	+	HAs
8078	M2	IP2+EVA	+	congenital	8	+	CI RE 3 y.o.; CI LE 2 y.o.
13358	M1+CEVA	EVA	–	6	23	–	CI LE 23 y.o.
13362	M1+CEVA	EVA	–	10	29	–	CI RE 31 y.o.
13392	M0	EVA	–	8	17	–	HAs
13393	M0	EVA	–	congenital	13	+	CI RE 14 y.o.; CI LE 23 y.o.
13887	M0	EVA+other *	–	10	18	–	HAs
4319	M2	IP2+EVA	+	1.5	10	+	CI RE 20 y.o.
11262	M2	IP2+EVA	+	congenital	5	–	CI RE 7 y.o.

Key: y.o., years old; PTA, pure-tone audiometry; HAs, hearing aids; CI, cochlear implant; RE, right ear; LE, left ear; +, present; –, absent; IP2, incomplete partition type 2; EVA, enlarged vestibular aqueduct; * cochlear hypoplasia type 4; ** asymmetric EVA.

## Data Availability

The data that support the findings of this study are available from the corresponding author upon request.
